# A new example of viral intein in Mimivirus

**DOI:** 10.1186/1743-422X-2-8

**Published:** 2005-02-11

**Authors:** Hiroyuki Ogata, Didier Raoult, Jean-Michel Claverie

**Affiliations:** 1 Information Génomique et Structurale, UPR2589 CNRS, IBSM, IFR88, 31 chemin Joseph Aiguier, 13402 Marseille Cedex 20, France; 2 Unité des Rickettsies, CNRS UPRESA 6020, Faculté de Médecine, 27 Boulevard Jean Moulin, 13385 Marseille Cedex 05, France

## Abstract

**Background:**

Inteins are "protein introns" that remove themselves from their host proteins through an autocatalytic protein-splicing. After their discovery, inteins have been quickly identified in all domains of life, but only once to date in the genome of a eukaryote-infecting virus.

**Results:**

Here we report the identification and bioinformatics characterization of an intein in the DNA polymerase PolB gene of amoeba infecting Mimivirus, the largest known double-stranded DNA virus, the origin of which has been proposed to predate the emergence of eukaryotes. Mimivirus intein exhibits canonical sequence motifs and clearly belongs to a subclass of archaeal inteins always found in the same location of PolB genes. On the other hand, the Mimivirus PolB is most similar to eukaryotic Polδ sequences.

**Conclusions:**

The intriguing association of an extremophilic archaeal-type intein with a mesophilic eukaryotic-like PolB in Mimivirus is consistent with the hypothesis that DNA viruses might have been the central reservoir of inteins throughout the course of evolution.

## Background

Mimivirus is the largest known virus, both in particle size (>0.4 μm in diameter) and genome length, recently discovered in amoeba, following the inspection of a hospital cooling tower prompted by a pneumonia outbreak [1]. Recently, its entire 1.2-Mbp genome sequence was determined [2]. Extensive phylogenetic studies and gene content analyses defined Mimivirus as a new family of nucleocytoplasmic large DNA viruses (NCLDV) besides *Poxviridae*, *Iridoviridae*, *Phycodnaviridae *and *Asfarviridae*, and suggested its early origin, probably before the individualization of the three domains of life [2].

While analyzing Mimivirus genome sequence, we noticed the unusual length of its putative DNA polymerase. A detailed analysis identified an intein in this gene. After the recent discovery of an intein in *Chilo *iridescent virus [3], an insect-infecting NCLDV of *Iridoviridae*, this is the second report of an intein sequence in a eukaryote-infecting virus.

Inteins are "protein introns" that catalyze self-splicing at the protein level. The splicing is defined by the self-catalytic excision of an intervening sequence ("intein") from a precursor host protein where it is located, and the concomitant ligation of the flanking amino- and carboxy-terminal fragments ("exteins") of the precursor. Inteins often possess a homing endonuclease domain, and are considered as mobile elements. Since their first discovery in 1990 [4,5], inteins have been identified in a wide variety of organisms, including bacteria, archaea, and unicellular eukaryotes, albeit with sporadic distribution (see  for a comprehensive list). For instance, they are relatively abundant in some hyperthermophilic archaea species (such as *Methanococcus jannaschii *possessing nineteen inteins), but absent in closely related species such as *Methanococcus maripaludis *[6]. Similarly, they are observed in many unrelated bacterial clades, but appear often limited to several species within each clade. It was suggested that viruses were potential "vectors" of inteins across species and responsible for the sporadic distribution of inteins [3]. Accordingly, inteins have been identified in many bacteriophages and prophages [7-10]. To our knowledge, the sole published account of eukaryote-infecting viruses harboring an intein concerns iridoviruses [3].

## Results

### Eukaryotic Polδ-like Mimivirus PolB

**Figure 1:**
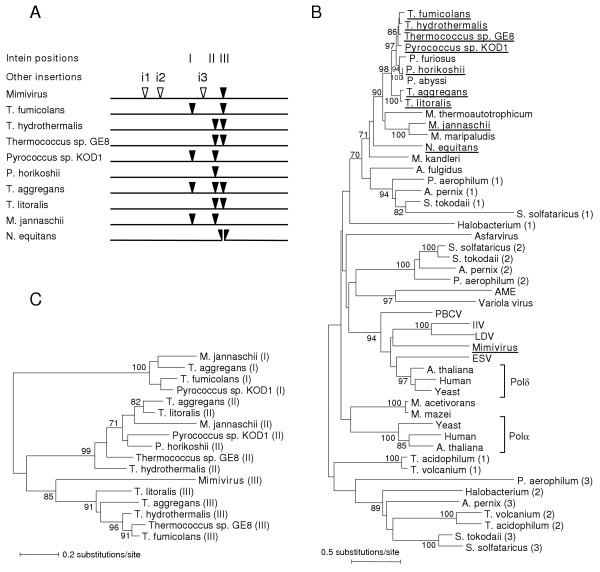
**(A) **Locations of inteins found in different DNA polymerases of the family B (PolB) (I, II, III; filled triangles) and other extra segments identified in the Mimivirus PolB (i1, i2, i3; open triangles). *Nanoarchaeum equitans *PolI is encoded in two pieces of genes (NEQ068, NEQ528), the break point of which corresponds to the position III intein integration site. Full intein motifs are comprised of the C-terminal part of NEQ068 and N-terminal part of NEQ528. **(B) **A phylogenetic tree of the family B DNA polymerases (PolBs) from diverse organisms, including Mimivirus (R322; GenBank AY653733), Paramecium bursaria Chlorella virus 1 (PBCV), Ectocarpus siliculosus virus (ESV), Invertebrate iridescent virus 6 (IIV), Lymphocystis disease virus 1 (LDV), Amsacta moorei entomopoxvirus (AME), Variola virus, Asfarvirus, eukaryotic DNA polymerase α and δ catalytic subunits, and archaeal DNA polymerase I. Intein containing genes are indicated by bold letters in the figure. Numbers in parentheses on the right of species name designate the numbering of paralogs. Sequences corresponding to inteins or Mimivirus extra segments (i1, i2, i3) were removed for the tree reconstruction. *N. equitans *PolI split genes were concatenated. **(C) **A phylogenetic tree based on the intein sequences found in PolBs. Numbers (I, II, and III) in parentheses on the right of species names indicate the intein integration sites. In (B) and (C), trees were built using a neighbor joining method, and rooted by the mid-point method. Bootstrap values larger than 70% are indicated along the branches.

Mimivirus genome sequence exhibits a putative ORF (R322, 1740 amino acid long) corresponding to a family B DNA polymerase PolB. This ORF R322 exhibits high scoring sequence homology (BLAST E-value<10^-24^) against eukaryotic PolBs in the public database. However, this Mimivirus PolB is much larger than its eukaryotic and viral homologues (about 1000 aa), and its optimal alignment with the other PolB sequences reveals four unmatched extraneous segments (Fig. 1A, Fig.  S1). Focusing on these extra segments, we identified a 351-aa intein (position 1053 to 1403) in the Mimivirus PolB sequence.

After removing those four Mimivirus specific insertions, the Mimivirus PolB sequence exhibited the highest BLAST scores (E-value = 10^-125^, 32% identity) against a soybean DNA polymerase Polδ (SWISS-PROT: O48901) with an alignment covering both the entire Mimivirus and the target sequence. Near equivalent matches are observed with a variety of eukaryotic (from yeast to human) family B DNA polymerase sequences. The best viral homologues were found in phycodnaviruses (E-value = 10^-116^). Conserved carboxylate residues (aspartate and glutamate) at the exonuclease and polymerase active sites [11,12] were all identified in the Mimivirus PolB (Fig.  S1). There was no other ORF encoding a putative PolB in the genome. These suggest that R322 encodes a functional PolB. Consistent with the homology search result, a phylogenetic analysis places the Mimivirus PolB near the root of eukaryotic Polδs (Fig. 1B). A similar branching position is obtained for the seven universally conserved Mimivirus genes [2]. Despite low bootstrap values for some of the deep branches in the Fig. 1B, this tree clearly indicates the lack of any specific affinity between the Mimivirus PolB and the archaeal PolB sequences containing inteins (bold letters in the Fig. 1B). It should also be noted that several other large DNA viruses are known to possess PolBs with a similar phylogenetic pattern [13].

### Canonical/archaeal type Mimivirus intein

**Figure 2:**
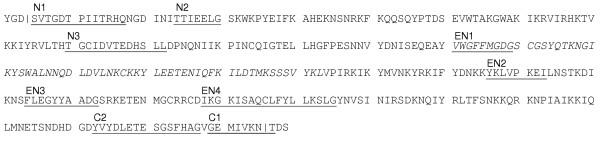
The Mimivirus DNA polymerase PolB intein. The 351 amino acid residues intein sequence is shown with, respectively, the last and the first three amino acid residues of the N-extein and the C-extein. Bold letters represent amino acid residues essential for protein splicing. Conserved intein sequence motifs are indicated by underlines (N1, N2, N3, EN1, EN2, EN3, EN4, C2 and C1). The sequence part matching to the Pfam LAGLIDADG endonuclease domain (PF00961, E-value = 0.16) is indicated by italic letters. The intein/extein boundaries are shown by '|'.

The Mimivirus intein sequence (351 aa) exhibits significant sequence similarities to several known inteins (E-value<10^-4^), all of which are from thermophilic/halophilic archaea. The best matching intein (E-value = 3 × 10^-8^) is the second intein of the *Thermococcus sp*. PolB (InBase: Tsp-GE8 Pol-2) with 24% amino acid sequence identity. The Mimivirus sequence exhibits all the expected features required for an active intein (Fig. 2). Sequence motifs [14] characterizing the splicing domain (N1-4, C2, C1) and the dodecapeptide LAGLIDADG homing-endonuclease domain (EN1-4) were all identified in the Mimivirus sequence except N4 motif. N4 motif is occasionally absent in the previously characterized active inteins [14]. Amino acid residues providing nucleophilic groups in self-splicing reactions are all present: the first serine and the last asparagine residues of the intein, and the first threonine residue of the downstream extein. Accordingly the Mimivirus intein is a canonical "asparagine-type" intein, of which the close homologues have previously been observed only in archaea species. In contrast, the previously reported *Chilo *iridescent virus intein is a non-canonical "glutamine-type" exhibiting a glutamine residue at the C-terminus [3,15]. The threonine and histidine residues in the N3 motif assisting in the initial acyl rearrangement at the N-terminal splice junction are also conserved. Thus, we predict that the Mimivirus intein is an active intein capable of self-splicing. The presence of a homing endonuclease domain suggests that this intein also retained its capacity to spread to other sites of the genome or to other organisms.

Other three inserts that we identified in the Mimivirus PolB are rather short. Those inserts are unique to Mimivirus, being not found in other PolB sequences. One of the extra segments of 197 aa found at the position 'i3' (Fig. 1A) exhibits a marginal sequence similarity to an intein within the replication factor C of *Methanococcus jannaschii *(E-value = 0.002, Fig. S2). However, it also exhibits a comparable level of sequence similarities to several unrelated database sequences, apparently containing low complexity sequences. The i3-insert lacks sequence features required for an active intein. The remaining two extra segments (88 and 121 aa at the position 'i1' and 'i2', respectively) did not exhibit any significant similarity to known protein sequences. The biological properties of those three Mimivirus specific inserts remain to be characterized.

### Mimivirus intein belongs to a specific allele type

**Figure 3:**
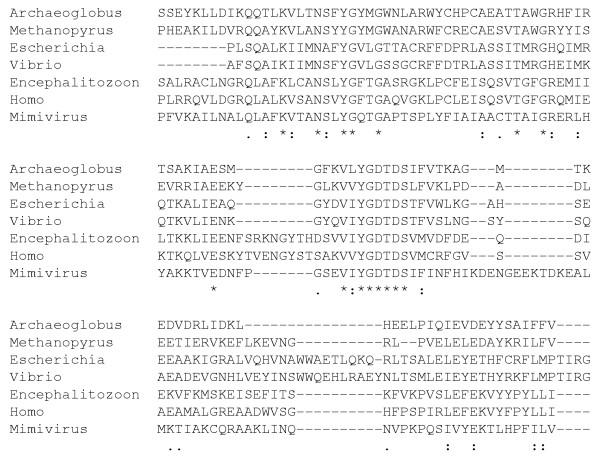
Sequence alignment of Family B DNA polymerases from the Archaea, Bacteria and Eukarya domains. The Mimivirus PolB sequence was used without its intein sequence. Only the region of the alignment around Mimivirus intein insertion site ("YGD|TDS") is shown. The insertion site precisely coincides with the most conserved positions in the sequences, as indicated by bold letters. This is the sole region in the entire sequence exhibiting 6 consecutive identical residues among PolB of the Archaea, Bacteria and Eukarya domains. SWISS-PROT/TrEMBL IDs are DPOL_ARCFU (*Archaeoglobus fulgidus*), Q8TWJ5 (*Methanopyrus kandleri*), DPO2_ECOLI (*Escherichia coli*), Q87NC2 (*Vibrio parahaemolyticus*), Q8SQP5 (*Encephalitozoon cuniculi*), and DPOD_HUMAN (Human).

Inteins have been identified in different types of DNA polymerases [16]. DNA polymerase catalytic subunits known to contain inteins are archaeal PolI, archaeal DNA polymerase II (PolII), bacterial DNA polymerase III α subunit (DnaE) and bacteriophage DNA polymerase I. Among these, archaeal PolI belongs to the family B DNA polymerase. Archaeal PolI contains up to three intein alleles, the insertion of which always occurs at one of three strictly conserved positions (I, II and III in Fig. 1A). Interestingly, the location of the bipartite inteins that separate the two PolI gene pieces of *Nanoarchaeum equitans *[17] coincides with position III. Remarkably, Mimivirus intein is exactly located at the position III (Fig. 1A). The sequence around the insertion site is highly conserved among different PolBs from evolutionary distant organisms such as *Escherichia coli *and human (Fig. 3). The crystal structure of *Pyrococcus kodakaraensis *PolI [11] reveals that those three distinct sites are in close spatial proximity, in the middle of the DNA binding domain and active site.

Perler *et al*. observed that inteins present in the same location within homologous genes ("intein alleles") tend to be more similar with each other than with inteins in different locations of the same gene or in different genes [18]. This phenomenon appears not only the simple consequence of regular vertical transmission of inteins, but also the result of lateral acquisitions through "homing" [19] at the same site of highly similar genes (i.e. "alleles") by the mechanism involving gene conversion [18]. Remarkably, the Mimivirus PolB intein holds this rule. The Mimivirus intein exhibits higher sequence homology scores to inteins at the position III of archaeal PolI (designated as "pol-c allele") than to inteins in the other PolI locations (I, II) or inteins in other genes. A phylogenetic analysis of the Mimivirus intein and other PolI inteins also supports the classification of the Mimivirus intein in this specific "intein allele"-type (Fig. 1C). This underlines the presence of intein subclasses ("intein alleles") each exhibiting its own preference of harboring site, even in such distantly related homologous genes such as Mimivirus PolB and archaeal PolI. It is implausible that the intein homing mechanism involving gene conversion have led to the direct transfer of an intein between such distantly related homologous genes. Nucleotide sequences (18 bp) around the pol-c allele insertion site do not exhibit unexpectedly high level of sequence similarities between Mimivirus (TATGGAGAC/ACGGACTCA for the amino acid sequence YGD/TDS) and archaeal sequences. For instance, the sequences from *M. jannaschii *and *Pyrococcus horikoshii *exhibit 7-missmaches (TATATTGAC/ACTGATGGA; MJ0885) and 5 mismatches (TATATAGAC/ACGGATGGA; PH1947), respectively. To the best of our knowledge, no evidence has been reported for a homing endonuclease recognizing such different sequences, although homing endonucleases are known to be rather tolerant of single-base-pair changes in their lengthy DNA recognition sequences [19]. A similar observation has been reported for DnaB inteins of *Rhodothermus marinus *and *Synechocystis *sp. PCC6803 [20].

A shift in the base compositions between intein and extein coding sequences is considered as indicating a recent acquisition of inteins [20]. Mimivirus PolB extein/intein DNA sequence compositions do not show a significant difference. Both exhibit similar G+C-contents (29%) and codon usages. In contrast, *Thermococcus fumicolans *PolI coding DNA (GenBank: Z69882) exhibits a G+C-content of 57% for the extein regions, compared to G+C-contents of 47% and 49% for its two inteins.

## Discussion

Archaeal PolI inteins have been described only in extremophiles, growing under conditions of temperature over 80°C (hyperthermophiles) or of high salinity (10 times that of sea water; halophiles). Mimivirus is mesophilic, growing in amoeba under the temprature of 37°C. The association of an archaeal-seqeunce-like intein with a eukaryotic-like PolB in Mimivirus thus suggests an indirect interaction between mesophilic eukaryotic viruses and extremophilic archaeabacteria. Mesophilic euryarchaea species similar to the methanogens associated with rumen [21,22] or related species found in human beings [23] might have mediated the transition of inteins between extreme environment and moderate one in the course of evolution. However, no data are available yet on the presence of inteins in the PolB genes of such mesophilic archaebacteria.

Lateral transfer (homing) might be responsible for the phylogenetic incongruence between inteins and exteins, and the same intein locations within homologues of distantly related organisms such as Mimivirus and archaea. However, given the specificity of homing endonucleases to long recognition sequences (12–40 bp) and the low level DNA sequence similarity between viral and archeal PolB homologues, a single recent homing event appears quite unlikely. The spread of inteins is better explained by a series of transfers, where inteins progressively accommodated small changes in their homing recognition sequences while retaining their gene position specificity. Such a cascade of transfers could have been mediated by DNA viruses [3]. Consistent results now start to accumulate including recent identification of several inteins in different iridoviruses (S. Pietrokovski pers. comm.), and an intein in a golden brown alga-infecting virus HaV of the *Phycodnaviridae *[24]. Given the similar base compositions of Mimivirus intein and extein, the low level of intein homology between Mimivirus and archaea, and the likely early origin of the Mimivirus/NCLDV lineage [2], it is tempting to speculate that these DNA viruses might have acquired inteins very early on, and acted as their central reservoir disseminating inteins across different domains of life in the long course of evolution.

## Conclusions

We have characterized a new viral intein found in the eukaryotic-type putative DNA polymerase PolB of Mimivirus by binformatics methods. The conservation of the active site motifs for splicing as well as its insertion at a catalytically important site of the PolB sequence suggests that the intein is most likely to be functional. Our phylogenetic analyses revealed that the intein sequence is closest to extremophilic archaeal inteins. The intriguing association of an extremophilic archaeal-type intein with a mesophilic eukaryotic-like PolB in Mimivirus is consistent with the hypothesis that DNA viruses might have been the central reservoir of inteins throughout the course of evolution.

## Methods

Sequence homology searches were carried out with the use of the BLAST programs [25] against the SWISS-PROT/TrEMBL database [26] and the New England Biolabs Intein Database [InBase, ; [Perler, 2002 #1380]]. Pfam [27] searches were carried out with the use of its web site . Multiple sequence alignments were generated with the use of T-Coffee [28]. Intein sequence motifs were identified through the inspection of a multiple intein sequence alignment. Neighbor joining tree analyses were conducted with the use of MEGA version 2.1 [29]. All the gap containing columns in multiple sequence alignments were removed before phylogenetic tree analyses. The gamma distance was applied to compute evolutionary distances. The gamma shape parameter (alpha) was estimated using the GZ-GAMMA program [30].

The sequence and annotation data for the Mimivirus PolB and intein was deposited to GenBank (accession number: AY606804). The complete genome sequence of Mimivirus is also available at GenBank (accession number: NC_006450). For a comprehensive description of the Mimivirus complete genome sequence and preliminary characterizations of the viral particle, see [2].

## Competing interests

The author(s) declare that they have no competing interests.

## Authors' contribution

HO carried out most of the sequence analysis, contributed to the interpretation of the results, and drafted the manuscript. DR contributed to the interpretation of the results. JMC contributed to the construction of the sequence alignment, participated in the interpretation of the results and finalized the manuscript.

## Supplementary Material

Additional File 1 **Supplementary figure S1 **Sequence alignment of Mimivirus PolB and eukaryotic Polδs. The Mimivirus intein sequence is removed, and its insertion site is highlighted by amino acid residues in red corresponding to the left three and right three resides around the insertion site. Three Mimivirus specific inserts (i1, i2 i3) were highlighted by blue letters. Conserved carboxylate residues in the exonuclease and polymerase active sites are highlighted by green background. Eukaryotic sequences were *Encephalitozoon cuniculi *(TrEMBL/SWISS-PROT: Q8SQP5), *Schizosaccharomyces pombe *(P30316) and *Glycine max *(soybean, O48901). Sequence alignment was obtained with the use of T-Coffee. Click here for file

Additional File 2 **Supplementary figure S2 **Sequence alignment of Mimivirus insert i3 and known intein sequences. Intein sequences are from *Methanococcus jannaschii *replication factor C (Mja RFC-3) and *Pyrococcus abyssi *replication factor C (Pab RFC-2). Click here for file

## References

[B1] La Scola B, Audic S, Robert C, Jungang L, de Lamballerie X, Drancourt M, Birtles R, Claverie JM, Raoult D (2003). A giant virus in amoebae. Science.

[B2] Raoult D, Audic S, Robert C, Abergel C, Renesto P, Ogata H, La Scola B, Suzan M, Claverie JM (2004). The 1.2-megabase genome sequence of Mimivirus. Science.

[B3] Pietrokovski S (1998). Identification of a virus intein and a possible variation in the protein-splicing reaction. Curr Biol.

[B4] Hirata R, Ohsumk Y, Nakano A, Kawasaki H, Suzuki K, Anraku Y (1990). Molecular structure of a gene, VMA1, encoding the catalytic subunit of H(+)-translocating adenosine triphosphatase from vacuolar membranes of Saccharomyces cerevisiae. J Biol Chem.

[B5] Kane PM, Yamashiro CT, Wolczyk DF, Neff N, Goebl M, Stevens TH (1990). Protein splicing converts the yeast TFP1 gene product to the 69-kD subunit of the vacuolar H(+)-adenosine triphosphatase. Science.

[B6] Hendrickson EL, Kaul R, Zhou Y, Bovee D, Chapman P, Chung J, Conway de Macario E, Dodsworth JA, Gillett W, Graham DE, Hackett M, Haydock AK, Kang A, Land ML, Levy R, Lie TJ, Major TA, Moore BC, Porat I, Palmeiri A, Rouse G, Saenphimmachak C, Soll D, Van Dien S, Wang T, Whitman WB, Xia Q, Zhang Y, Larimer FW, Olson MV, Leigh JA (2004). Complete genome sequence of the genetically tractable hydrogenotrophic methanogen Methanococcus maripaludis. J Bacteriol.

[B7] van der Wilk F, Dullemans AM, Verbeek M, van den Heuvel JF (1999). Isolation and characterization of APSE-1, a bacteriophage infecting the secondary endosymbiont of Acyrthosiphon pisum. Virology.

[B8] Lazarevic V (2001). Ribonucleotide reductase genes of Bacillus prophages: a refuge to introns and intein coding sequences. Nucleic Acids Res.

[B9] Pedulla ML, Ford ME, Houtz JM, Karthikeyan T, Wadsworth C, Lewis JA, Jacobs-Sera D, Falbo J, Gross J, Pannunzio NR, Brucker W, Kumar V, Kandasamy J, Keenan L, Bardarov S, Kriakov J, Lawrence JG, Jacobs WRJ, Hendrix RW, Hatfull GF (2003). Origins of highly mosaic mycobacteriophage genomes. Cell.

[B10] Ward N, Larsen O, Sakwa J, Bruseth L, Khouri H, Durkin AS, Dimitrov G, Jiang L, Scanlan D, Kang KH, Lewis M, Nelson KE, Methe B, Wu M, Heidelberg JF, Paulsen IT, Fouts D, Ravel J, Tettelin H, Ren Q, Read T, Deboy RT, Seshadri R, Salzberg SL, Jensen HB, Birkeland NK, Nelson WC, Dodson RJ, Grindhaug SH, Holt I, Eidhammer I, Jonasen I, Vanaken S, Utterback T, Feldblyum TV, Fraser CM, Lillehaug JR, Eisen JA (2004). Genomic Insights into Methanotrophy: The Complete Genome Sequence of Methylococcus capsulatus (Bath). PLoS Biol.

[B11] Hashimoto H, Nishioka M, Fujiwara S, Takagi M, Imanaka T, Inoue T, Kai Y (2001). Crystal structure of DNA polymerase from hyperthermophilic archaeon Pyrococcus kodakaraensis KOD1. J Mol Biol.

[B12] Doublie S, Tabor S, Long AM, Richardson CC, Ellenberger T (1998). Crystal structure of a bacteriophage T7 DNA replication complex at 2.2 A resolution. Nature.

[B13] Villarreal LP, DeFilippis VR (2000). A hypothesis for DNA viruses as the origin of eukaryotic replication proteins. J Virol.

[B14] Pietrokovski S (1998). Modular organization of inteins and C-terminal autocatalytic domains. Protein Sci.

[B15] Amitai G, Dassa B, Pietrokovski S (2004). Protein splicing of inteins with atypical glutamine and aspartate C-terminal residues. J Biol Chem.

[B16] Perler FB (2002). InBase: the Intein Database. Nucleic Acids Res.

[B17] Waters E, Hohn MJ, Ahel I, Graham DE, Adams MD, Barnstead M, Beeson KY, Bibbs L, Bolanos R, Keller M, Kretz K, Lin X, Mathur E, Ni J, Podar M, Richardson T, Sutton GG, Simon M, Soll D, Stetter KO, Short JM, Noordewier M (2003). The genome of Nanoarchaeum equitans: insights into early archaeal evolution and derived parasitism. Proc Natl Acad Sci U S A.

[B18] Perler FB, Olsen GJ, Adam E (1997). Compilation and analysis of intein sequences. Nucleic Acids Res.

[B19] Belfort M, Roberts RJ (1997). Homing endonucleases: keeping the house in order. Nucleic Acids Res.

[B20] Liu XQ, Hu Z (1997). A DnaB intein in Rhodothermus marinus: indication of recent intein homing across remotely related organisms. Proc Natl Acad Sci U S A.

[B21] Tajima K, Nagamine T, Matsui H, Nakamura M, Aminov RI (2001). Phylogenetic analysis of archaeal 16S rRNA libraries from the rumen suggests the existence of a novel group of archaea not associated with known methanogens. FEMS Microbiol Lett.

[B22] Whitford MF, Teather RM, Forster RJ (2001). Phylogenetic analysis of methanogens from the bovine rumen. BMC Microbiol.

[B23] Kulik EM, Sandmeier H, Hinni K, Meyer J (2001). Identification of archaeal rDNA from subgingival dental plaque by PCR amplification and sequence analysis. FEMS Microbiol Lett.

[B24] Nagasaki K, Shirai Y, Tomaru Y, Nishida K, Pietrokovski S (2005). Algal viruses with distinct intraspecies host specificities include identical intein elements.. Appl Environ Microbiol.

[B25] Altschul SF, Madden TL, Schaffer AA, Zhang J, Zhang Z, Miller W, Lipman DJ (1997). Gapped BLAST and PSI-BLAST: a new generation of protein database search programs. Nucleic Acids Res.

[B26] Boeckmann B, Bairoch A, Apweiler R, Blatter MC, Estreicher A, Gasteiger E, Martin MJ, Michoud K, O'Donovan C, Phan I, Pilbout S, Schneider M (2003). The SWISS-PROT protein knowledgebase and its supplement TrEMBL in 2003. Nucleic Acids Res.

[B27] Bateman A, Birney E, Cerruti L, Durbin R, Etwiller L, Eddy SR, Griffiths-Jones S, Howe KL, Marshall M, Sonnhammer EL (2002). The Pfam protein families database. Nucleic Acids Res.

[B28] Notredame C, Higgins DG, Heringa J (2000). T-Coffee: A novel method for fast and accurate multiple sequence alignment. J Mol Biol.

[B29] Kumar S, Tamura K, Jakobsen IB, Nei M (2001). MEGA2: molecular evolutionary genetics analysis software. Bioinformatics.

[B30] Gu X, Zhang J (1997). A simple method for estimating the parameter of substitution rate variation among sites. Mol Biol Evol.

