# Supplementary material for: A new example of viral intein in Mimivirus
Source: Virol J. 2005 Feb 11;2:8. doi: 10.1186/1743-422X-2-8 (PMC549080; doi:10.1186/1743-422X-2-8)
Supplement: Additional File 1 — Supplementary figure S1 Sequence alignment of Mimivirus PolB and eukaryotic Polδs. The Mimivirus intein sequence is removed, and its insertion site is highlighted by amino acid residues in red corresponding to the left three and right three resides around the insertion site. Three Mimivirus specific inserts (i1, i2 i3) were highlighted by blue letters. Conserved carboxylate residues in the exonuclease and polymerase active sites are highlighted by green background. Eukaryotic sequences were Encephalitozoon cuniculi (TrEMBL/SWISS-PROT: Q8SQP5), Schizosaccharomyces pombe (P30316) and Glycine max (soybean, O48901). Sequence alignment was obtained with the use of T-Coffee. [file 1743-422X-2-8-S1.pdf]

```
E.cuniculi      ------------------------------------------------------------
S.pombe         MTDRSSNEGVVLNKENYPFPRRNGSIHGEITDVKRRRLSERNGYGDKKGSSSKEKTSSFE
Soybean         ----------------------MTQEEEFMDEDVFINETLVSEDEESLILRDIEQRQALA
Mimivirus_PolB  -----------------------------------------------MPSETIDSTKQFE


E.cuniculi      -----------------------------MENEIVFCQTHIDFYMDDSGLSYSCPVFSIF
S.pombe         DELAEYASQLDQDEIKSSKDQQWQRPALPAINP-EKDDIYFQQIDSEEFTEGSVPSIRLF
Soybean         NRLSKWTRP------PLSAGYVAQSRSVLFQQLEIDYVIAESHGELLPNSSGPVAIIRIF
Mimivirus_PolB  FQISDWNSYHELDQEEEEK-----------------------------------YVIQLF
                                                                        : :*

E.cuniculi      GNTKSGRPVRVLARNFFPYFYVEPSNGKEYKEEDIKESVQRLDVKAT-------------
S.pombe         GVTDNGNSILVHVVGFLPYFYVKAPVG--FRPEMLERFTQDLD----------ATCNGGV
Soybean         GVTKEGHSVCCNVHGFEPYFYICCPPG--MGPDDISHFHQTLEGRM-----REANRNSNV
Mimivirus_PolB  GRTEDDHDVCLKVTGYTPFFYVEIPKQ--WKQRQVDKFVEILKNKVQYHCKKNLDEDFDL
                * *.... :   . .: *:**:  .         :..  : *.

E.cuniculi      ---ILEVEAVMKQSILGYTEGKTRVY-RLTLNTPHVSTALKVLLESGI-SVK---GEKV-
S.pombe         IDHCI---IEMKENLYGFQGNEKSPFIKIFTTNPRILSRARNVFERGEFNFEELFPVGV-
Soybean         GKFVRRIEMVQRRSIMYYQQSNSQPFLKIVVALPTMVASCRGILDRGI----QLDGLGMK
Mimivirus_PolB  SKSLIKYAMVKKHKFYNFRNKQLYNFLLLVFKSHTAMKEFSSILARPL-EAKGLTNKPM-
                           :..:  :   :   :  :             ::              :

E.cuniculi      RFRVYESNFPFVLRFMCDLGIVGMSYLRV--NRYE-----ILDADHLTVSASYESLESLP
S.pombe         GVTTFESNTQYLLRFMIDCDVVGMNWIHLPASKYQFRYQNRVSNCQIEAWINYKDLISLP
Soybean         SFLTYESNVLFALRFMIDCNIVGGNWIGIPAGKYK-KTAKSLSYCQLEFDCLYSELISHA
Mimivirus_PolB  LYQRYESNIEPHIRFMHINNLSSCGWASIDKDKLK-KIPE-YSNCDYSFSVNWKDV---K
                    :***    :***   .: . .:  :  .: :       .  .      :..:

E.cuniculi      LEGGYVVLPPLKVLSIDIECVSAGNGFP--SSKCDPIIQIGNTLMLFGDNSYASQDIFCL
S.pombe         AEGQWSKMAPLRIMSFDIECAGRKGVFP--DPSIDPVIQIASIVTQYGDSTPFVRNVFCV
Soybean         PEGEYSKMAPFRILSFDIECAGRKGHFP--EPTHDPVIQIANLVTLQGEDQPFIRNVMTL
Mimivirus_PolB  PSNNDDRMAPFKIMGYDIECVSCDQNFPQAERPSDKIIQIGITMYRYGSMKCYEQHILTL
                 ..    :.*::::. ****..    **  .   * :***.  :   *.     :.:: :
                                Exonuclease active site

E.cuniculi      KETTGIPGVNVHWYETEKELLESWKKYFMELDPDVIVGYNVKGFDIPYILSRGEILGIES
S.pombe         DTCSQIVGTQVYEFQNQAEMLSSWSKFVRDVDPDVLIGYNICNFDIPYLLDRAKSLRIHN
Soybean         KSCSPIVGVDVMPFETEREVLLAWRDFIREVDPDIIIGYNICKFDLPYLIERALNLKIAE
Mimivirus_PolB  KKCAPIEGVNVECYKKEKGLLRGFAKKIAELRPDFKTGYNNFGFDDKYIYDRILRIDKRE
                .  : * *.:*  ::.:  :* .: . . :: **.  ***   **  *: .*   :   .

E.cuniculi      ---------------------------------------FSVLGRSRKK-ARTRDTT---
S.pombe         ---------------------------------------FPLLGRIHNFFSVAKETT---
Soybean         ---------------------------------------FPILGRIRNSRVRVKDTT---
Mimivirus_PolB  GKKQGVNINALKNKFMDEILRTIGKVNNNYLIENEGLDRIPIYTTVKDKKISSKASRFIQ
                                                       :.:   :.      : :
                <=(i1:88aa)=================================================

E.cuniculi      ----------------------------MSSNMFGSITTTEVEIDGRLIIDMMVVIRRDF
S.pombe         ----------------------------FSSKAYGTRESKTTSIPGRLQLDMLQVMQRDF
Soybean         ----------------------------FSSRQYGTRESKEVAVEGRVTFDLLQVMQRDY
Mimivirus_PolB  IRGGTYVENGNNLKYVQSPGITYFEVKNLSSSALGDNELKFIQIPGVLSIDMMKVIQRDH
                                            :**   *    .   : * : :*:: *::**.
                ===========================>

E.cuniculi      KLRSYSLNSVSIHFLKEQ------------------------------------------
S.pombe         KLRSYSLNAVCSQFLGEQ------------------------------------------
Soybean         KLSSYSLNSVSSHFLSEQ------------------------------------------
Mimivirus_PolB  RLIGYKLDNVSANFITEKADKIIEMPHNQEDSDSEKEDEDTDDKTYDVNIYTKSTKALEK
                :* .*.*: *. :*: *:
                                  <=(i2:121aa)==============================
```

```
E.cuniculi        ------------------------------------------------------------
S.pombe           ------------------------------------------------------------
Soybean           ------------------------------------------------------------
Mimivirus_PolB    DSYIQIMVNDGYSSSPLSEGAKYKVYDIQTITEKKLNEKTNKEEIFVYQAIKTKICQKDI

                  ============================================================

E.cuniculi        -------------------KEDVPYSSIGE-LQSKNKDTRRRIASYCLRDTVLPLRLFNT
S.pombe           -------------------KEDVHYSIITD-LQNGTADSRRRLAIYCLKDAYLPQRLMDK
Soybean           -------------------KEDVHHSIISD-LQNGNAETRRRLAVYCLKDAYLPQRLLDK
Mimivirus_PolB    QQLRETIKNPLLGISWTFAKDDMHHTKINEYFEEGDPKKIRQIAKYCLKDCKLVNLLLAK
                                     *:*: :: * : ::.   .. *::* ***:*  *   *: .
                  ==================>

E.cuniculi        LNVLINYTELSRVTGTPIEYFFTRGMAIKIFTLVYRAASKEDFMIPDID------PFESN
S.pombe           LMCFVNYTEMARVTGVPFNFLLARGQQIKVISQLFRKALQHDLVVPNIR------VNGTD
Soybean           LMFIYNYVEMARVTGVPISFLLSRGQSIKVLSQLLRRARQKNLVIPNAK------QAGSE
Mimivirus_PolB    LEIIVNSVGMAKVCHVPLSYLFLRGQGVKIFSLVSKKCREKNFLIPVLRRKSKDNEGDED
                  *  : * . :::*  .*:.::: **  :*::: : : . :.::::*             :

E.cuniculi        K-TFEGGFVIEPRKGFYNKPVSVMDFSSLYPSIMISHNLCYTTLLTKEQYRILG------
S.pombe           E-QYEGATVIEPIKGYYDTPIATLDFSSLYPSIMQAHNLCYTTLLDSNTAELLKLKQ---
Soybean           QGTFEGATVLEARAGFYEKPIATLDFASLYPSIMMAYNLCYCTLVIPEDARKLNIPP---
Mimivirus_PolB    E-TYEGATVITPKPNVYLSPIGVLDYSSLYPNSMRERNLSQECYVDDS--KYDNLPGYIY
                  :  :**. *: .  . * .*:..:*::****. *   **.    :  .  .
                                          (intein insertion site, I)       <==
                                          Polymerase active site

E.cuniculi        ------------------------------------------------------------
S.pombe           ----------------DVD-----------------------------------------
Soybean           ----------------E-S-----------------------------------------
Mimivirus_PolB    HDVEIILKDKKGKILRNIDGTPQKEYHRFAQEIITDEQINRELKDIFDKINTVFENNVAI

                  =(i3:197aa)=================================================

E.cuniculi        ------------------------------------------------------------
S.pombe           ------------------------------------------------------------
Soybean           ------------------------------------------------------------
Mimivirus_PolB    IQNQKYFTEKNISELIDKHKNISDSKIEDIEFDESLSDKRKNKLVDAEKDSLDKNIGFYQ

                  ============================================================

E.cuniculi        ------------------------------------------------------------
S.pombe           ------------------------------------------------------------
Soybean           ------------------------------------------------------------
Mimivirus_PolB    KIKSQIDKIKLDSKIEIDNLSKNLNEEEKSKQINKMELNTKNLISKVFSKYLITEQQREE

                  ============================================================

E.cuniculi        --------------GTKTPTGNYFCSAERKKGLLPRILTDLLTSRKRIKEELEREKDSAL
S.pombe           --------------YSVTPNGDYFVKPHVRKGLLPIILADLLNARKKAKADLKKETDPFK
Soybean           --------------VNRTPSGETFVKSNLQKGILPEILEELLTARKRAKADLKEAKDPLE
Mimivirus_PolB    LIVLEKERAKRSVNAEKAKVYNTVDGITVRYGILPEILTELLNKRKETNGKLANEKDPFV
                                   :   : .     : *:** ** :**. **. : .* . .*.
                  =============>

E.cuniculi        RACLNGRQLAFKLCANSLYGFTGASRGKLPCFEISQSVTGFGREMIILTKKLIEENFSRK
S.pombe           KAVLDGRQLALKVSANSVYGFTGATNGRLPCLAISSSVTSYGRQMIEKTKDVVEKRYRIE
Soybean           KAVLDGRQLALKISANSVYGFTGATIGQLPCLEISSSVTSYGRQMIEHTKKLVEDKFTTL
Mimivirus_PolB    KAILNALQLAFKVTANSLYGQTGAPTSPLYFIAIAACTTAIGRERLHYAKKTVEDNFP--
                  :* *:. ***:*: ***:** ***. . *  : *: ..*. **: :  :*. :*..:
                               (intein insertion site, II)
```

```
E.cuniculi      NGYTHDSVVIYGDTDSVMVDFDEQ-----------DIEKVFKMSKEISEFITSKFVKPVS
S.pombe         NGYSHDAVVIYGDTDSVMVKFGVK-----------TLPEAMKLGEEAANYVSDQFPNPIK
Soybean         NGYEHNAEVIYGDTDSVMVQFGVS-----------AVEEAMNLGREAAEHISGTFTKPIK
Mimivirus_PolB  -----GSEVIYGDTDSIFINFHIKDENGEEKTDKEALMKTIAKCQRAAKLINQNVPKPQS
                     .: ********:::.*  .            : :.:   .. :: :.  . :* .
```

(intein insertion site, III)

Polymerase active site

```
E.cuniculi      LEFEKVYYPYLLINKKRYAGLLYSNPENPSKIDTRGIETVRRDNCRLVKEVVETVLEMIL
S.pombe         LEFEKVYFPYLLISKKRYAGLFWTRTDTYDKMDSKGIETVRRDNCPLVSYVIDTALRKML
Soybean         LEFEKVYYPYLLISKKRYAGLFWTKPDNFDKMDTKGIETVRRDNCLLVKNLVNDCLHKIL
Mimivirus_PolB  IVYEKTLHPFILVAKKKYVGLLFEKSPDKYFLKSMGIVLKRRDNAPIVKIVVGGIIDNIL
                : :**. .*::*: **:*.**:: ..     :.: **   ****. :*. ::   :  :*

E.cuniculi      YQKNVEKAKQFVKDAVRDLYLGRTDLSLLVISKSLTKAGDKYESKQAHVQLAEKLRKRDE
S.pombe         IDQDVEGAQLFTKKVISDLLQNKIDMSQLVITKALSKT--DYAAKMAHVELAERMRKRDA
Soybean         IDRDIPGAVQYVKNAISDLLMNRMDLSLLVITKGLTKTGDDYEVKAAHVELAERMRKRDA
Mimivirus_PolB  KNRDIDKAIEYTKIVLDKLMNGEYPMDKFIISKTL-KSRYKKPSTIAHKVLADRMAVRDP
                 ::::  *  :.* .: .*  ..  :. ::*:* * *:  .   . **  **:::  **

E.cuniculi      STAPVLGDRVPYVIVRKEKGAAAH------EKSEDPVYVLENNLPIDTEYYISQQISKPL
S.pombe         GSAPAIGDRVAYVIIKGAQGDQFY------MRSEDPIYVLENNIPIDAKYYLENQLSKPL
Soybean         ATAPNVGDRVPYVIIKAAKGAKAY------ERSEDPIYVLENNIPIDPHYYLENQISKPI
Mimivirus_PolB  GNKPQINDRIPFVYIVKDMGKKKKDILQGDLIEHPEYVIANNLKIDYLYYLEHQIINPA
                .. * :.**:.:* :    *             *.* **: **: **  **:.:*: :*

E.cuniculi      SRIFEPIMDNV--QELFRGDHTR-IAASTGLKGPMNTFLKPTDTCVG---CRAEGRI-IC
S.pombe         LRIFEPILGEK-ASSLLHGDHTRTISMAAPSVGGIMKFAVKVETCLGCKAPIKKGKTALC
Soybean         LRIFEPILKNA-SKELLHGSHTRSISISTPSNSGILRFAKKQLPALVVKLYLARVITLSV
Mimivirus_PolB  SQILELMMDTKDVQKFFN----KYIIDEQNKRKGAQSLTK--------------------
                 :*:* ::     ..::.    : *            :

E.cuniculi      INCVK-----------------------DFHVHLQKMQREVEDKKEKLNSCWVECQRCQ
S.pombe         ENCLNRSAEL-----------------------YQRQVAQVNDLEVRFARLWTQCQRCQ
Soybean         HIAKEGRLSCTVKQYLKCLSWRCFLGGCGHSVRSAKVHFIRMFSAPVGIVQFSIDEKRHR
Mimivirus_PolB  --------WMDFSKLPKESGSKTAKKPYQSQKLQKTKSSNKSQIDPKYINLIKNKSRKHE
                                                        .       :     . :: .

E.cuniculi      GSIHNQVLCVNRDCPIFYMRTKVKKELVPLNDRLRKLRSFDW
S.pombe         GSMHQDVICTSRDCPIFYMRIAEHKKLQQSVDLLKRFDEMSW
Soybean         KIWVKQSCNWTDGTSKFCQEFDLADLFEPMDTNTIWCLPQS-
Mimivirus_PolB  CQNMNKWISSTDKCTDDWEPIVE-------------------
                    :.    .   .
```
