# Supplementary material for: A new example of viral intein in Mimivirus
Source: Virol J. 2005 Feb 11;2:8. doi: 10.1186/1743-422X-2-8 (PMC549080; doi:10.1186/1743-422X-2-8)
Supplement: Additional File 2 — Supplementary figure S2 Sequence alignment of Mimivirus insert i3 and known intein sequences. Intein sequences are from Methanococcus jannaschii replication factor C (Mja RFC-3) and Pyrococcus abyssi replication factor C (Pab RFC-2). [file 1743-422X-2-8-S2.pdf]

```
Mja_RFC-3       CLTGDAKITLPDEREIKIEDFIKMFEERKLKHVLNRNGEDLVLAGVKFNSKIVNHKVYRL
Pab_RFC-2       CVTGDTKVYTPDEREVKIRDFMNYFENGLIKEVSNRIGRDTVIAAVSFNSRIVGHPVYRL
Mimivirus_i3    ------------------------------------------------------------


Mja_RFC-3       VLESGREIEATGDHKFLTRDGWKEVYELKEDDEVLVYPALEGVGFEVDERRIIGLNEFYE
Pab_RFC-2       TLESGRIIEATGDHMFLTPEGWKQTYDIKEGSEVLVKPTLEGTPYEPDPRVIIDIKEFYN
Mimivirus_i3    ------------------------------------------------------------


Mja_RFC-3       FLTNYEIKLGYKPLGKAKSYKELITRDKEKILSRVLELSDKYSKS---------------
Pab_RFC-2       FLEKIEREHNLKPLKEAKTFRELITKDKEKILRRALELRAEIENGLTKREAEILELISAD
Mimivirus_i3    ---------------YIYHDVEIILKDKK-------------------------------
                                     *:* :**:

Mja_RFC-3       --------------------------------------------------------EIRR
Pab_RFC-2       TWIPRAELEKKARISRTRLNQILQRLEKKGYIERRIEGRKQFVRKIRNGKILRNAMDIKR
Mimivirus_i3    -----------------------------GKILRNIDGTPQ-------------------


Mja_RFC-3       KIEEEFGIKISLTTIKNLINGKIDGFALKYVRKIKELGWDEITYDDEKAGIFARLLGFII
Pab_RFC-2       ILEEEFGIKISYTTVKKLLSGNVDGMAYRILKEVKEKWL--VRYDDEKAGILARVVGFIL
Mimivirus_i3    -------------------------------------------------KEYHRFAQEII
                                                                     *.   *:

Mja_RFC-3       GDGHLSKSKEGRILITATINELEGIKKDLEKLGIK----ASNIIEKDIEHKLDGREIKGK
Pab_RFC-2       GDGHLARN--GRIWFNSSKEELEMLANDLRKLGLK----PSEIIERDSSSEIQGRKVKGR
Mimivirus_i3    TDEQINRE--LKDIFDKINTVFENNVAIIQNQKYFTEKNISELIDK--------------
                 * :: :.   :  :      :*     :.:         *::*::

Mja_RFC-3       TSFIYINNKAFYLLLNFWGVEIGNKTINGYNIPKWIKYGNKFVKREFLRGLFGADGTKPY
Pab_RFC-2       IYMLYVDNAAFHALLRFWKVEVGNKTKKGYTVPEWIKKGNLFVKREFLRGLFGADGTKPC
Mimivirus_i3    -----------------------HKNISDSKIEDIEFDESLSDKRK--------------
                                       :*. .. .: .     .   **:

Mja_RFC-3       IKKYNINGIKLGIRVENISKDKTLEFFEEVKKMLEEFEVESYIKVSKIDNKNLTEL-IVK
Pab_RFC-2       GKRYNFNGIKLEIRAKKESLERTVEFLNDVADLLREFDVDSKITVSPTKEGFIIRL-IVT
Mimivirus_i3    ---------NKLVDAEKDSLDKNIGFYQKIKSQIDKIKLDSKIEIDNL-SKNLNEEEKSK
                         :  : .:: * ::.: * :.: . : ::.::* * :.   .  : .    .

Mja_RFC-3       ANNKNYLKYLSRISYAYEKDNFARLVGEYLRIKEAYKDIILKEIAENALKEADGEKSLRE
Pab_RFC-2       PNDANYLNFLTRVGYAYAKDTYARLVGEYIRIKLAYKNIILPGIAEKAIELATVTNS-TY
Mimivirus_i3    QINKMELNTKNLISKVFSK-----------------------------------------
                  :   *:  . :. .: *

Mja_RFC-3       LARKYNVPVDFIINQLKGKDIGLPRNFMTFEEFLKEKVVDGKYVSERIIKKECIGYRDVY
Pab_RFC-2       AAKVLGVSRDFVVNRLKGTQIGITRDFMTFEEFMKERVLNG-YVIEKVIKKEKLGYLDVY
Mimivirus_i3    -------------------------------YLITEQQREELIVLEKERAKRSVN-----
                                                ::.*:  :   * *:   *. :.

Mja_RFC-3       DITCHKDPSFIANGFVSHN
Pab_RFC-2       DVTCARDHSFISNGLVSHN
Mimivirus_i3    -------------------
```
